# Assessment of Surgeon Variation in Adherence to Evidence-Based Recommendations for Treatment of Trigger Finger

**DOI:** 10.1001/jamanetworkopen.2019.12960

**Published:** 2019-10-11

**Authors:** Jessica I. Billig, Kelly A. Speth, Jacob S. Nasser, Lu Wang, Kevin C. Chung

**Affiliations:** 1 Veterans Affairs (VA)/National Clinician Scholars Program, VA Health Services Research and Development Center for Clinical Management Research, VA Ann Arbor Healthcare System, Ann Arbor, Michigan; 2 Section of Plastic Surgery, Department of Surgery, Michigan Medicine, Ann Arbor; 3 Department of Biostatistics, University of Michigan, Ann Arbor; 4 Comprehensive Hand Center, Section of Plastic Surgery, Michigan Medicine, Ann Arbor

## Abstract

**Importance:**

Stenosing tenosynovitis (trigger finger) affects approximately 2% of the population. Given the prevalence of trigger finger and rising health care costs, adherence to the cost-effective and evidence-based treatment algorithm will permit better outcomes and allocation of resources.

**Objectives:**

To examine treatment patterns for trigger finger and to determine surgeon-level and patient-level factors that influence adherence to evidence-based treatment.

**Design, Setting, and Participants:**

This retrospective population-based cohort study examined deidentified claims for treatment of trigger finger from a national insurance provider using the Clinformatics Data Mart database. Patients were included if they were 18 years or older and treated from January 1, 2002, through December 31, 2016 (excluding a washout period from July 1, 2008, until June 30, 2010), with a new diagnosis of single-digit trigger finger. Data were analyzed from December 21, 2018, through April 28, 2019.

**Exposures:**

Cost-effective and evidence-based research published in July 2009 for the treatment of trigger finger.

**Main Outcomes and Measures:**

After excluding the 1-year washout period on either side of July 1, 2009, adherence to the recommended treatment algorithm of 2 corticosteroid injections before surgical release of trigger finger was compared with practice before publication of research supporting this cost-effective and evidence-based approach.

**Results:**

In this analysis of 83 667 patients with trigger finger, 52 698 (63.0%) were women, and 20 045 (24.0%) had type 1 or 2 diabetes. Mean (SD) age was 61 (13) years. From 2002 to 2016, an overall increasing trend in adherence to the cost-effective and evidence-based approach to treatment was noted, with no significant increase in adherence in the postpublication era (67.5% vs 73.3%; *P* = .27). Substantial variation in adherence was observed at the surgeon level (intraclass correlation, 33%). Plastic surgeons had no change in adherence over time compared with orthopedic surgeons (odds ratio [OR], 1.00; 95% CI, 0.98-1.02; *P* = .90), whereas general surgeons had increased adherence (OR, 1.04; 95% CI, 1.02-1.06; *P* < .001). Higher-volume surgeons were also more adherent to these evidence-based recommendations (OR, 1.59; 95% CI, 1.53-1.65; *P* < .001).

**Conclusions and Relevance:**

This study found substantial surgeon-level variation in adherence to evidence-based treatment of trigger finger. Surgeon specialty and volume were associated with differences in adherence. Efforts to understand surgeon barriers to implementation, regardless of physician specialty, appear to be necessary, and better implementation strategies may permit increased uptake of evidence-based treatment of trigger finger.

## Key Points

**Question:**

What surgeon characteristics are associated with adherence to the evidence-based cost-effective approach for the treatment of stenosing tenosynovitis (trigger finger)?

**Findings:**

In this population-based cohort study of 83 667 patients with single-digit trigger finger, 33% of the variation in adherence was associated with surgeon-level characteristics, including specialty type and surgeon volume.

**Meaning:**

These findings suggest that understanding barriers to implementation of evidence-based treatment of trigger finger and improved implementation strategies should be a priority to improve outcomes and reduce costs.

## Teaser

This population-based cohort study examines treatment patterns for stenosing tenosynovitis (trigger finger) and determines surgeon-level factors associated with adherence to evidence-based treatment.

## Introduction

Evidence-based practices are associated with better treatment outcomes and patient satisfaction.^1,2^ Despite comprehensive recommendations from researchers and policy makers, physicians often fail to provide patients with proper treatment, resulting in detrimental care, wasted resources, and unnecessary spending.^3^ As health care costs in the United States continue to rise,^4^ policy makers are becoming increasingly interested in identifying variations in care to reduce discretionary spending and increase the quality of care. Furthermore, recent estimates reveal that only two-thirds of health care payments are based on high-value care.^5,6^ Thus, as the United States transitions from fee-for-service to value-based care, defining the lapses in care quality is imperative in health care reform.^7^

Stenosing tenosynovitis (trigger finger) is one of the most common hand conditions, with a prevalence of 2% in the general population.^8^ This condition is caused by inflammation and narrowing of the A1 pulley, often resulting in a painful locking and triggering when the finger is flexed.^9^ The treatment for trigger finger typically consists of corticosteroid injections and/or surgery to release the A1 pulley. Although surgical treatment provides patients with satisfactory outcomes, recent evidence suggests that conservative treatment modalities, specifically corticosteroid injections, should be attempted before surgery.^10,11,12,13^ In 2009, Kerrigan and Stanwix^14^ published a cost-minimization analysis to identify the least costly and most effective treatment algorithm for trigger finger. The Medicare reimbursement for a corticosteroid injection was approximately $171, and the cost for surgery ranged from $601 to $1203 depending on location.^14^ The researchers found that 2 corticosteroid injections before any surgical treatment offered the patients adequate relief while being the least costly option. Subsequent research has reinforced the efficacy of corticosteroid injections for trigger finger and recommendations of 2 injections before release.^10,11,14^ Despite these recommendations, adherence at a national level is not yet known. Given the high prevalence of trigger finger, a comprehensive understanding of the treatment variations may help guide clinical decisions and aid in the allocation of resources for this patient population.

As health care costs in the United States continue to rise, it becomes increasingly important to provide physicians with guidance regarding cost-effective treatment. The purpose of this study is to investigate national adherence to an evidence-based approach for treatment of trigger finger and thereby determine avenues to provide high-value care for these patients. We used a national sample of individuals treated for single-digit trigger finger to examine adherence to the cost-effective treatment algorithm. Our specific objectives are to (1) identify trends of adherence to a commonly accepted and cost-effective treatment algorithm and (2) examine the role of surgeon- and patient-level factors associated with adherence.

## Methods

### Study Population

We used the Clinformatics Data Mart database (OptumInsight) from January 1, 2002, through December 31, 2016, to conduct our study. Clinformatics Data Mart contains inpatient and outpatient deidentified patient encounters for more than 140 million enrollees covered by private insurance. This study received exempt status from the University of Michigan’s institutional review board, which waived the need to obtain informed consent because the data were deidentified. We followed the Strengthening the Reporting of Observational Studies in Epidemiology (STROBE) reporting guidelines.

We included patients 18 years or older with a new diagnosis of single-digit trigger finger. To obtain our cohort, we identified patients using *International Classification of Diseases, Ninth Revision, Clinical Modification* (*ICD-9-CM*) and *International Statistical Classification of Diseases and Related Health Problems, Tenth Revision* (*ICD-10*) diagnosis codes (eTable 1 in the Supplement). We ensured that all patients were continuously enrolled in the database 1 year before their initial trigger finger diagnosis to confirm a new diagnosis. In addition, all patients had at least 1 treatment for trigger finger within 1 year after diagnosis. Patients were excluded as having evidence of multiple trigger fingers if they had more than 1 specific trigger finger *ICD-9-CM* or *ICD-10* code or more than 1 *Current Procedural Terminology* code related to a treatment of trigger finger during an encounter to isolate single-digit diagnoses. Given the nature of claims data and the broad specificity of *ICD-9-CM* codes and some *ICD-10* codes, for some patients it was ambiguous whether more than 1 trigger finger was treated. To ensure that the appropriate cohort was evaluated, sensitivity analyses were performed. To capture treatment data, we ensured that all patients had at least 1 year of continuous enrollment in the database after their initial diagnosis. We also restricted the physicians to those who could perform the whole range of interventions, including corticosteroid injections and surgery. The eFigure in the Supplement illustrates the inclusion and exclusion characteristics. Data were analyzed from December 21, 2018, through April 28, 2019.

### Outcome

Our primary outcome was adherence to the evidence-based and cost-effective treatment approach of 2 corticosteroid injections before undergoing A1 pulley release.^10,11,14,15^ This treatment recommendation was provided by Kerrigan and Stanwix^14^ in July 2009. Their report was the first cost-minimization analysis using data from the literature that revealed the cost-effectiveness of 2 corticosteroid injections before surgical release. To allow for uptake of the recommendation, we used a washout period of 1 year before or after July 1, 2009. This washout period also ensured that all patients in the analysis were treated before or after the publication and not during both periods. Therefore, we defined the prepublication era as January 1, 2002, through June 30, 2008, and the postpublication era as July 1, 2010, through December 31, 2016. We considered adherence to the evidence-based approach as 1 to 2 corticosteroid injections without surgery or 2 corticosteroid injections before undergoing surgery (eTable 2 in the Supplement). All other combinations of treatment were deemed nonadherence.

### Explanatory Variables

Variables of interest included patient-level and surgeon-level characteristics. We collected demographic patient data including age, sex, Elixhauser comorbidity score, presence of type 1 or 2 diabetes with and without complications, and geographic location. The Elixhauser comorbidity score was calculated for each patient as a proxy for health status using *ICD-9-CM* and *ICD-10* codes.^16^ Surgeon-level characteristics included specialty, surgeon-level volume, and geographic location. Surgeon specialty encompassed general, plastic, and orthopedic surgery because these 3 specialties are eligible for subspecialty certification in surgery of the hand.^17^

### Statistical Analysis

We conducted descriptive analyses of adherence to the evidence-based approach to the treatment of trigger finger. We estimated 95% CIs to investigate trends and variability in visit-level adherence using bootstrap resampling with 500 observations repeated 100 times. We then examined the association between patient- and surgeon-level characteristics with adherence over time. Given the nonrandom clustering of patient visits within surgeons, we used multilevel logistic regression with random intercepts at the surgeon level to assess surgeon-level variation in rates of adherence over time. Fixed slopes were used in the model. The covariates in the model consisted of patient age, a diagnosis of diabetes in the patient, time, surgeon specialty, and surgeon volume and included an interaction for the differential change in adherence by surgeon specialty over time. Model diagnostics revealed a satisfactory model fit. The intraclass correlation was also calculated to determine the percentage of variation at the surgeon level. To support the conclusions of our analysis, we performed a sensitivity analysis excluding all patients with a possibility of multiple trigger finger diagnoses based on *ICD-9-CM* and *ICD-10* codes. Finally, given recent evidence showing the efficacy of 3 corticosteroid injections before release,^18^ we performed an additional sensitivity analysis with these treatments as adherent regimens. The significance level was set at 2-sided *P* < .05 for all analyses. Analyses were performed using SAS software, version 9.4 (SAS Institute Inc) and R, version 3.5.1 (R Project for Statistical Computing).

## Results

A total of 83 667 patients (30 969 men [37.0%] and 52 698 women [63.0%]; mean [SD] age, 61 [13] years) received treatment for single-digit trigger finger from 2002 through 2016 by 11 120 different surgeons with 110 012 patient visits. The surgeons included 9837 orthopedic surgeons (88.5%), 869 plastic surgeons (7.8%), and 414 general surgeons (3.7%). The median number of procedures performed per surgeon was 3 (interquartile range, 1-8). Of all patients included in the final cohort, the mean (SD) age in the prepublication era was 57 (12) years compared with 63 (13) years in the postpublication era. In the total cohort, 20 045 (24.0%) had a diagnosis of diabetes. Compared with the prepublication era, an increase in diabetes was seen in the postpublication era (4762 [19.3%] vs 12 462 [26.4%]). Table 1 illustrates the overall patient-level characteristics and stratification by timing.

**Table 1.** Patient-Level Characteristics

Characteristic	Patient Group^a^		
	Overall (N = 83 667)	Prepublication Era (n = 24 722)^b^	Postpublication Era (n = 47 223)^c^
Age, mean (SD), y	61 (13)	57 (12)	63 (13)
Sex, No. (%)			
Male	30 969 (37.0)	8518 (34.5)	18 044 (38.2)
Female	52 698 (63.0)	16 204 (65.5)	29 179 (61.8)
Diabetes, No. (%)	20 045 (24.0)	4762 (19.3)	12 462 (26.4)
Elixhauser comorbidity score, mean (SD)^d^	2.2 (2.1)	1.7 (1.8)	2.4 (2.2)

^a^ Washout period accounts for a total of 11 722 patients.

^b^ Includes January 1, 2002, through June 30, 2008.

^c^ Includes July 1, 2010, through December 31, 2016.

^d^ Scores range from 0 to 19, with higher scores indicating greater number of comorbidities.

**Figure 1.**
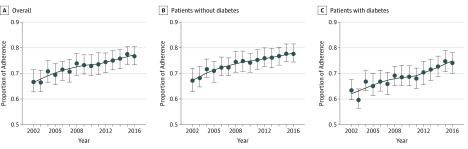
Mean Adherence to Evidence-Based Treatment of Trigger Finger Error bars indicate 95% CIs obtained from bootstrapped, resampled patients with 500 observations repeated 100 times. The line represents a nonparametric, locally estimated smoothing to view the adherence trend over time.

Table 2 outlines the adherence to the evidence-based approach in the prepublication and postpublication eras for all patients and those with and without diabetes. In the prepublication era, 22 093 visits (67.5%) were adherent compared with 45 189 (73.3%) in the postpublication era, but this difference was not statistically significant (*P* = .27). Among patients with diabetes, 4006 (62.7%) had adherence in the prepublication era compared with 11 245 (69.5%) in the postpublication era. Figure 1A displays the increase in adherence to the overall treatment regimen over time, and Figure 1B and C show the increase stratified by patients without and with diabetes.

**Table 2.** Adherence to Evidence-Based Treatment Over Time

Adherence	No./Total No. (%) by Patient Group^a^	
	Prepublication Era^b^	Postpublication Era^c^
Visits with total adherence	22 093/32 736 (67.5)	45 189/61 669 (73.3)
Diabetes		
With	4006/6391 (62.7)	11 245/16 182 (69.5)
Without	18 087/26 345 (68.7)	33 944/45 487 (74.6)
Surgeons		
Orthopedic	19 879/29 303 (67.8)	39 577/54 288 (72.9)
Plastic	1191/1881 (63.3)	2581/3569 (72.3)
General	1023/1552 (65.9)	3031/3812 (79.5)

^a^ Washout period accounts for a total of 15 607 visits and includes a total of 110 012 visits.

^b^ Includes January 1, 2002, through June 30, 2008.

^c^ Includes July 1, 2010, through December 31, 2016.

**Figure 2.**
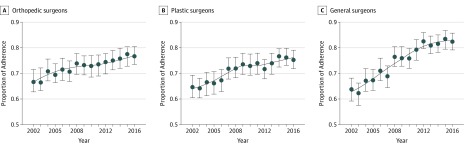
Mean Adherence to Evidence-Based Treatment of Trigger Finger Stratified by Surgeon Includes 94 559 visits for orthopedic surgeons, 6069 visits for plastic surgeons, and 5947 visits for general surgeons. Error bars indicate 95% CIs obtained from bootstrapped, resampled patients with 500 observations repeated 100 times. The line represents a nonparametric, locally estimated smoothing to view the adherence trend over time.

Figure 2 displays adherence over time stratified by surgeon type. After adjusting for patient-level and surgeon-level characteristics, general surgeons were significantly less adherent than orthopedic surgeons (odds ratio [OR], 0.66; 95% CI, 0.41-0.92; *P* = .002), but a larger differential increase in adherence occurred over time among general surgeons relative to orthopedic surgeons (OR, 1.04; 95% CI, 1.02-1.06; *P* < .001). Plastic surgeons had no change in adherence over time compared with orthopedic surgeons (OR, 1.00; 95% CI, 0.98-1.02; *P* = .90). In addition, higher-volume surgeons had higher odds of adherence than lower-volume surgeons (OR, 1.59; 95% CI, 1.53-1.65; *P* < .001). For patients with diabetes, odds of adherence had a statistically significant decrease (OR, 0.74; 95% CI, 0.70-0.78; *P* < .001). Table 3 illustrates our multilevel model with surgeon-level and patient-level characteristics. Approximately 33% of the variation in care was explained by the surgeon-level characteristics.

**Table 3.** Multilevel Logistic Regression Model of Adherence

Variable	OR (95% CI)	*P* Value
Time	1.04 (1.04-1.04)	<.001
Diabetes	0.74 (0.70-0.78)	<.001
Age	0.99 (0.98-1.01)	.31
Surgeon		
Orthopedic	1 [Reference]	NA
Plastic	0.84 (0.63-1.04)	.09
General	0.66 (0.41-0.92)	.002
Surgeon × time^a^		
Orthopedic surgeon	1 [Reference]	NA
Plastic surgeon	1.00 (0.98-1.02)	.90
General surgeon	1.04 (1.02-1.06)	<.001
Surgeon volume^b^	1.59 (1.53-1.65)	<.001

Abbreviations: NA, not applicable; OR, odds ratio.

^a^ Denotes the interaction term for surgeon and time.

^b^ Given its nonnormal distribution, surgeon volume was calculated on the log_10_ scale.

Sensitivity analyses using a smaller subset of patients with trigger finger and no possibility of multiple trigger finger diagnoses demonstrated similar results, although the adherence rates were slightly higher (15 325 [69.7%] in the prepublication era and 32 260 [74.4%] in the postpublication era) (eTable 3 in the Supplement). Also, general surgeons had overall adherence similar to that of orthopedic surgeons (OR, 0.76; 95% CI, 0.46-1.05; *P* = .06) (eTable 4 in the Supplement). In the multilevel model, the variation explained at the surgeon level was approximately 33%. Additional sensitivity analyses including 3 corticosteroid injections before release revealed similar adherence rates (22 200 [67.8%] in the prepublication era and 45 245 [73.3%] in the postpublication era) (eTable 5 in the Supplement), with 32% of the variation of care explained by surgeon-level characteristics (eTable 6 in the Supplement).

## Discussion

In this study, we observed an increase over time in adherence to an evidence-based treatment algorithm for trigger finger with no significant increase in uptake after the publication of this treatment approach. Substantial variation in adherence occurred over time, with 33% of the variation attributable to surgeon-level characteristics. General surgeons had an increase in adherence over time to these evidence-based recommendations compared with orthopedic surgeons. Further, surgeons treating more patients with trigger finger had increased odds of following evidence-based recommendations. These findings suggest a need for better implementation strategies in the treatment of trigger finger to improve outcomes at a lower cost.

With increasing research and growing evidence for specific treatments, a drive to implement clinically effective and cost-effective care has occurred. However, delays in the translation of evidence-based approaches into everyday practice continue.^3,19^ In our study, even with a washout period of 1 year to allow for surgeon uptake of evidence, only 73.3% of patient treatment plans adhered to the cost-effective and evidence-based recommendations outlined in the publication by Kerrigan and Stanwix^14^ and reinforced by additional research.^10,11^ In addition, no substantial increase in adherence occurred after these publications. These findings reinforce the need for better implementation strategies at the surgeon level to improve adherence to evidence-based practices. Using the consolidated framework for advancing implementation science, specific constructs can help guide effective implementation of evidence-based treatment of trigger finger. These constructs may include identifying surgeon characteristics that promote implementation, such as knowledge of the evidence-based practice, and engaging surgeons in the adoption of these practices.^20^ For trigger finger, in which surgeon-level factors account for 33% of the variation in treatment adherence, better strategies may be needed to increase awareness of evidence-based approaches and to enhance behavioral change in surgeons. Possible strategies to increase awareness may include dissemination of guidelines to all surgeons and their associated organizations regardless of surgical specialty, creation of more stringent continuing education programs, and establishment of regional and national quality collaboratives to measure adherence and provide feedback to all stakeholders in a noncompetitive environment. For example, in trying to implement a surgical checklist for safe surgery, consensus implementation strategies included surgical checklist implementation champions, education on how best to use the surgical checklist, integration of the checklist into already established systems for each specific hospital, and engagement and support of the team to permit staff adoption of the checklist.^21,22,23,24^ However, implementation strategies must be tailored to fit each specific behavioral change effort, and implementation strategies for the treatment of trigger finger are lacking.

The development of high-level evidence regarding the optimal treatment algorithm for patients with trigger finger may guide the implementation of uniform trigger finger care. Given national initiatives to improve the quality of health care, identifying variation, especially at the surgeon level, and devising policies to reduce this variation is vital. Our results reveal that surgeon type and surgeon volume were associated with adherence to the cost-effective and evidence-based approach to the treatment of trigger finger. For surgeon-level variation, a thorough understanding of the barriers and facilitators for implementation may help improve outcomes and reduce cost.^20,25^ In a study by Gotlib Conn et al,^26^ the implementation of an enhanced recovery-after-surgery protocol for elective colorectal surgery was facilitated by the establishment of a surgeon champion, developing relationships with a bottom-up approach, and building visibility of the program within the institution. These facilitators were associated with successful uptake and adherence to the enhanced recovery-after-surgery protocol.^26^ Once the barriers and facilitators are determined, implementation strategies targeted at altering surgeon behavior may lead to improvement in adherence to evidence-based approaches.^27,28^ For example, Goldberg et al^29^ investigated barriers to physician adherence to evidence-based monitoring guidelines in leukemia and found that resource barriers, lack of familiarity, and lack of agreement prevented the adoption of evidence into practice. Nevertheless, research examining the factors associated with increased uptake of clinically based guidelines and recommendations for surgical subspecialties is lacking. Given the differences in adherence based on surgeon-level characteristics, it appears that identifying specific barriers and facilitators is the next step in improving adherence to evidence-based medicine. We believe that future qualitative research interviewing surgeons from all specialties that treat trigger finger is warranted to improve the integration of evidence into practice.

Surgeon variation in practice patterns may result from multiple factors, including patient preferences. Studies assessing the desire for shared decision-making in hand surgery have shown that patients prefer to actively participate in the surgical decision-making process.^30^ In a study by Dardas et al^30^ of older adults with hand conditions, 81% of patients desired a more patient-directed role in the decision-making process. Moreover, studies have shown a disconnect between patient and surgeon priorities for treatment of other hand conditions.^31^ However, in a study by Hawley et al^32^ assessing determinants of surgeon variation for breast cancer treatment, similar patients, based on clinical factors such as tumor stage, received different treatment depending on their surgeon, highlighting the influence of the surgeon on the decision-making process for treatment. Therefore, providing patients with treatment options based on current evidence and improving patients’ knowledge of the risks and benefits of these options may limit surgeon variation and reduce decisional conflict for patients.^31,33^ Surgeon variation in treatment is likely multifactorial, and a combined approach of increasing implementation strategies to disseminate evidence-based medicine and incorporating evidence into shared decision-making may decrease this variation.

High-level evidence regarding outcomes for treatment of trigger finger is needed before the development of implementation strategies and clinical practice guidelines. The current evidence typically consists of single-center, retrospective studies. However, some small-scale randomized clinical trials assessing corticosteroid injections vs surgery for trigger finger have been performed.^12,34,35^ Findings reveal the superior long-term efficacy of surgery compared with corticosteroid injections. However, corticosteroid injections have a success rate of 50% to 90% depending on the trial.^12,34,35^ Nonetheless, these trials do not account for complication profiles, ease of administration, cost, and additional patient burdens, such as time off work, for the different treatment options. Therefore, a multicenter randomized clinical trial, in conjunction with other high-level prospective studies, is needed to inform surgeons and policy makers of the most clinically effective treatment for trigger finger. These studies should incorporate patient-reported outcomes and additional end points beyond relief of the trigger finger to capture the whole benefit of the different treatment options. Such evidence may help reinforce the need for clinical practice guidelines regarding trigger finger treatment.

Patient-level factors may also influence adherence to evidence-based medicine. In our study, patients with diabetes had less adherence to those without diabetes. However, we observed an overall increase in adherence for patients with diabetes over time. Diabetes may make trigger finger refractory to corticosteroid injections, and some surgeons may question the efficacy of these injections for patients with diabetes.^34,36,37^ A study by Luther et al^38^ examined the cost of corticosteroid injections and surgical release in patients with diabetes and found that immediate surgical release in patients with insulin-dependent diabetes is more cost-effective with the assumption of at least a 34% failure rate. However, the investigators limited their surgical releases in location, which may affect the cost-effectiveness of their study,^39^ and controversy exists surrounding the failure rate of corticosteroid injections for trigger finger among patients with diabetes. A single-center randomized clinical trial of patients undergoing corticosteroid injections for the treatment of trigger finger found that corticosteroid injections were significantly more effective in patients without diabetes, with success in 25 of 29 digits (86.2%) compared with 12 of 19 (63.2%) in patients with diabetes. Although these findings reveal the superior effectiveness of corticosteroid injections for patients without diabetes, patients with diabetes may still gain some benefit from corticosteroid injections.^34^ However, more robust data are needed to fully understand the role of corticosteroid injections in patients with diabetes to establish guidelines on the optimal treatment for trigger finger in these patients. Therefore, understanding when and how patient-level factors may affect adherence will help promote high-value health care and should be considered when developing metrics to examine adherence to evidence-based care.

### Limitations

As with other studies using administrative data, this study includes several limitations. We were unable to assess the clinical severity of the trigger finger, which may play a role in treatment decisions. Nonetheless, the research and recommendations do not account for severity of trigger finger, and surgeons should practice evidence-based medicine. We also controlled for diabetes, which, based on the literature, is the most common cause of failing conservative treatment.^34,36,37^ Other surgeon-level factors that were not included in our model might influence adherence. We also could not investigate the physician-patient interaction, which may influence treatment decisions, or whether the treatment decisions were surgeon or patient driven. We restricted our physician cohort to those who could perform surgery, and therefore we cannot comment on the adherence of health care professionals who are not surgeons. Additionally, this study uses diagnosis codes that are for billing purposes, which may be subject to misclassification.

## Conclusions

Our study illustrates that adherence to an evidence-based approach to the treatment of trigger finger increased over time but not specifically after the publication of important research. Surgeon variation in adherence was present with specialty and volume, accounting for a substantial amount of this variation. With national incentives to promote quality in health care, these findings suggest that implementation of evidence-based approaches for the treatment of trigger finger should be a priority.

## Notes

Supplement. **eTable 1.** Diagnosis and Procedure Codes **eTable 2.** Adherence to Evidence-Based Treatment Algorithm **eTable 3.** Sensitivity Analysis of Adherence to Evidence-Based Treatment Over Time With a Sample Excluding Patients With a Possibility of Multiple Trigger Fingers (76 123 Visits) **eTable 4.** Sensitivity Analysis of Multilevel Logistic Regression Model of Adherence With a Sample Excluding Patients With a Possibility of Multiple Trigger Fingers **eTable 5.** Sensitivity Analysis of Adherence to Evidence-Based Treatment Over Time Using a Modified Definition of Adherence to Allow for Up to 3 Corticosteroid Injections Before Release (110 012 Visits) **eTable 6.** Sensitivity Analysis of Multilevel Logistic Regression Model of Adherence Using a Modified Definition of Adherence to Allow for Up to 3 Corticosteroid Injections Before Release **eFigure.** Inclusion and Exclusion Criteria
