# Supplementary material for: Assessment of Surgeon Variation in Adherence to Evidence-Based Recommendations for Treatment of Trigger Finger
Source: JAMA Netw Open. 2019 Oct 11;2(10):e1912960. doi: 10.1001/jamanetworkopen.2019.12960 (PMC6804023; doi:10.1001/jamanetworkopen.2019.12960)
Supplement: Supplement. — eTable 1. Diagnosis and Procedure Codes eTable 2. Adherence to Evidence-Based Treatment Algorithm eTable 3. Sensitivity Analysis of Adherence to Evidence-Based Treatment Over Time With a Sample Excluding Patients With a Possibility of Multiple Trigger Fingers (76 123 Visits) eTable 4. Sensitivity Analysis of Multilevel Logistic Regression Model of Adherence With a Sample Excluding Patients With a Possibility of Multiple Trigger Fingers eTable 5. Sensitivity Analysis of Adherence to Evidence-Based Treatment Over Time Using a Modified Definition of Adherence to Allow for Up to 3 Corticosteroid Injections Before Release (110 012 Visits) eTable 6. Sensitivity Analysis of Multilevel Logistic Regression Model of Adherence Using a Modified Definition of Adherence to Allow for Up to 3 Corticosteroid Injections Before Release eFigure. Inclusion and Exclusion Criteria [file jamanetwopen-2-e1912960-s001.pdf]

Supplementary Online Content

Billig JI, Speth KA, Nasser JS, Wang L, Chung KC. Assessment of surgeon variation in adherence to evidence-based recommendations for treatment of trigger finger. *JAMA Netw Open.* 2019;2(10):e1912960. doi:10.1001/jamanetworkopen.2019.12960

eTable 1. Diagnosis and Procedure Codes

eTable 2. Adherence to Evidence-Based Treatment Algorithm

eTable 3. Sensitivity Analysis of Adherence to Evidence-Based Treatment Over Time With a Sample Excluding Patients with a Possibility of Multiple Trigger Fingers (76 123 Visits)

eTable 4. Sensitivity Analysis of Multilevel Logistic Regression Model of Adherence With a Sample Excluding Patients With a Possibility of Multiple Trigger Fingers

eTable 5. Sensitivity Analysis of Adherence to Evidence-Based Treatment Over Time Using a Modified Definition of Adherence to Allow for Up to 3 Corticosteroid Injections Before Release (110 012 Visits)

eTable 6. Sensitivity Analysis of Multilevel Logistic Regression Model of Adherence Using a Modified Definition of Adherence to Allow for Up to 3 Corticosteroid Injections Before Release

eFigure. Inclusion and Exclusion Criteria

This supplementary material has been provided by the authors to give readers additional information about their work.

© 2019 Billig JI et al. *JAMA Network Open*

eTable 1. Diagnosis and Procedure Codes

Coding System	Definition	Codes
International Classification of Disease, 9th Revision (ICD-9)	Trigger Finger	727.00, 727.03, 727.05
International Statistical Classification of Diseases and Related Health Problems, 10th Revision (ICD-10)	Trigger Finger	M65.3, M65.30, M65.31, M65.32, M65.33, M65.34, M65.35
Current Procedural Terminology Code	Steroid Injection of Tendon Sheath	20550
	Trigger Finger Release	26055

© 2019 Billig JI et al. *JAMA Network Open*

eTable 2. Adherence to Evidence-Based Treatment Algorithm

Treatment Algorithm	Category	Description	Evidence-based Adherence
0	Injections-only	1-2 codes for steroid injection without subsequent codes for surgery or steroid injections	Yes
1	Injections-only	3+ codes for steroid injections, without subsequent code for surgery after the second injection	No
2	Surgery-only	Only codes for trigger finger release	No
3	1 injection, then surgery	One code for steroid injection prior to trigger finger release	No
4	2 injections, then surgery*	Two codes for steroid injection prior to trigger finger release	Yes
5	3+ injections, then surgery*	Three or more codes for steroid injection prior to trigger finger release	No

© 2019 Billig JI et al. *JAMA Network Open*

eTable 3. Sensitivity Analysis of Adherence to Evidence-Based Treatment Over Time With a Sample Excluding Patients with a Possibility of Multiple Trigger Fingers (76 123 Visits)

**Adherence**	**Pre-Era***	**Post-Era***
Total Adherence, No. (%)	15,325 (70%)	32,260 (74%)
Patients with Diabetes, No. (%)	2,733 (65%)	7,930 (70%)
Patients without Diabetes, No. (%)	12,592 (71%)	24,330 (76%)
Orthopaedic Surgeons, No. (%)	13,744 (70%)	28,229 (74%)
Plastic Surgeons, No. (%)	830 (66%)	1,851 (74%)
General Surgeons, No. (%)	751 (70%)	2,180 (80%)

*Washout period accounts for a total of 10,790 visits
Pre-era: January 1, 2002- June 30, 2008
Post-era: July1, 2010- December 31, 2016

© 2019 Billig JI et al. *JAMA Network Open*

eTable 4. Sensitivity Analysis of Multilevel Logistic Regression Model of Adherence With a Sample Excluding Patients With a Possibility of Multiple Trigger Fingers

	**OR (95% CI)**	**P-value**
Time, year	1.04 (1.04-1.05)	<0.001
Diabetes	0.72 (0.68-0.77)	<0.001
Age	0.98 (0.96-1.00)	0.31
Surgeon		
Orthopaedic Surgeon	1[Ref]	
Plastic Surgeon	0.90 (0.66-1.14)	0.40
General Surgeon	0.76 (0.46-1.05)	0.06
Surgeon * Time[a]		
Orthopaedic Surgeon	1[Ref]	
Plastic Surgeon	1.00 (0.98-1.02)	0.99
General Surgeon	1.04 (1.01-1.06)	0.005
Surgeon Volume[b]	1.60 (1.54-1.67)	<0.001

[a]Denotes the interaction term for surgeon and time.
[b]Given its non-normal distribution, surgeon volume was calculated on the $\log_{10}$ scale.

© 2019 Billig JI et al. *JAMA Network Open*

eTable 5. Sensitivity Analysis of Adherence to Evidence-Based Treatment Over Time Using a Modified Definition of Adherence to Allow for Up to 3 Corticosteroid Injections Before Release (110 012 Visits)

**Adherence**	**Pre-Era***	**Post-Era***
Total Adherence, No. (%)	22,200 (68%)	45,245 (73%)
Patients with Diabetes, No. (%)	4,055 (63%)	11,253 (70%)
Patients without Diabetes, No. (%)	18,145 (69%)	33,992 (75%)
Orthopaedic Surgeons, No. (%)	19,981 (68%)	39,612 (73%)
Plastic Surgeons, No. (%)	1,187 (63%)	2,604 (73%)
General Surgeons, No. (%)	1,032 (66%)	3,029 (79%)

*Washout period accounts for a total of 15,607 visits
Pre-era: January 1, 2002- June 30, 2008
Post-era: July1, 2010- December 31, 2016

© 2019 Billig JI et al. *JAMA Network Open*

eTable 6. Sensitivity Analysis of Multilevel Logistic Regression Model of Adherence Using a Modified Definition of Adherence to Allow for Up to 3 Corticosteroid Injections Before Release

	**OR (95% CI)**	**P-value**
Time, year	1.04 (1.03-1.04)	<0.001
Diabetes	0.75 (0.72-0.79)	<0.001
Age	1.01 (0.99-1.02)	0.33
Surgeon		
Orthopaedic Surgeon	1[Ref]	
Plastic Surgeon	0.89 (0.68-1.10)	0.27
General Surgeon	0.75 (0.49-1.00)	0.03
Surgeon * Time[a]		
Orthopaedic Surgeon	1[Ref]	
Plastic Surgeon	1.00 (0.98-1.02)	0.78
General Surgeon	1.03 (1.01-1.05)	0.008
Surgeon Volume[b]	1.71 (1.65-1.77)	<0.001

[a]Denotes the interaction term for surgeon and time.
[b]Given its non-normal distribution, surgeon volume was calculated on the $\log_{10}$ scale.

© 2019 Billig JI et al. *JAMA Network Open*

eFigure. Inclusion and Exclusion Criteria

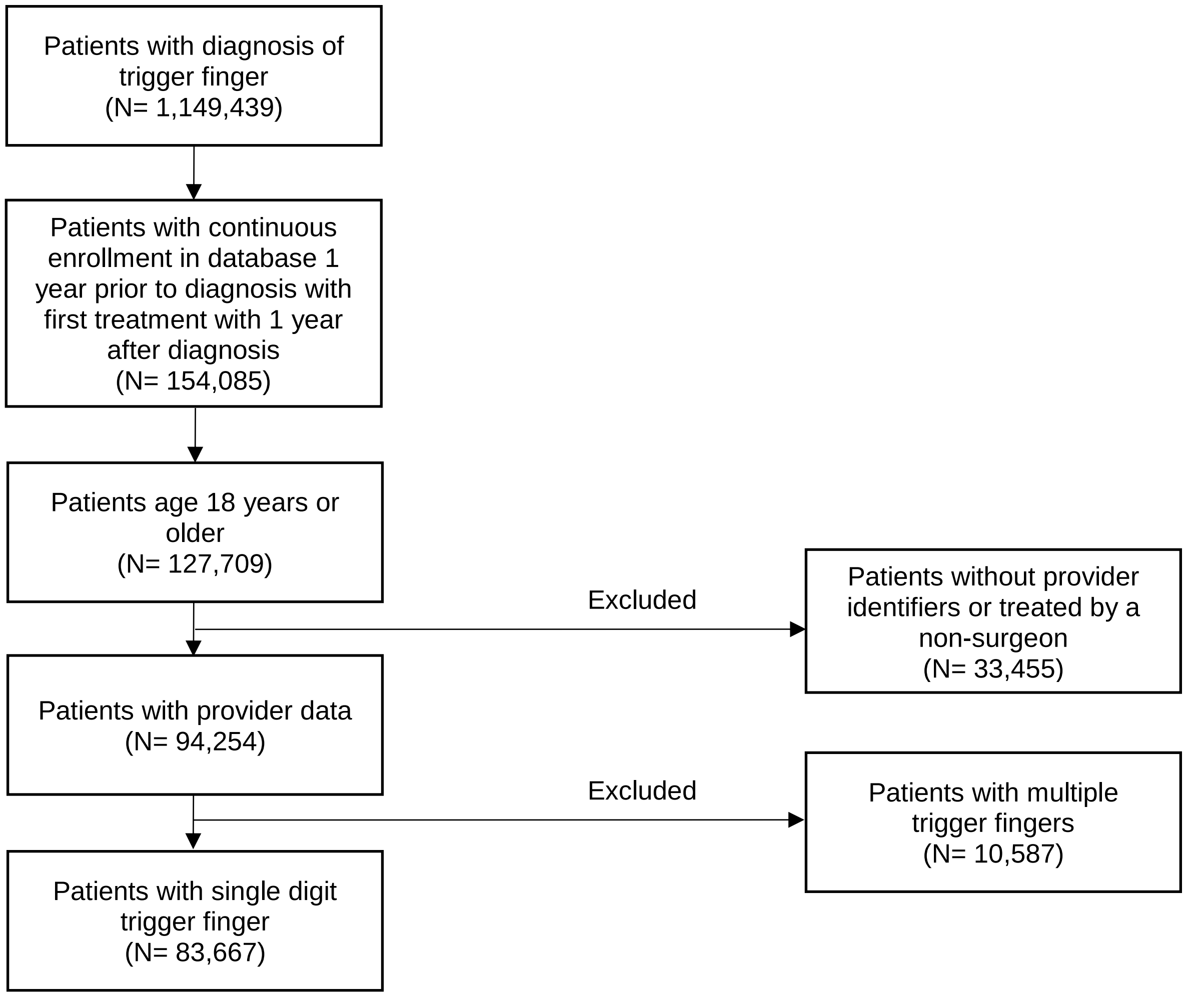

© 2019 Billig JI et al. *JAMA Network Open*
